# Driving-Related Neuropsychological Performance in Stable COPD Patients

**DOI:** 10.1155/2013/297371

**Published:** 2013-02-04

**Authors:** Foteini Karakontaki, Sofia-Antiopi Gennimata, Anastasios F. Palamidas, Theocharis Anagnostakos, Epaminondas N. Kosmas, Anastasios Stalikas, Charalambos Papageorgiou, Nikolaos G. Koulouris

**Affiliations:** ^1^Respiratory Function Laboratory, 1st Department of Respiratory Medicine, National University of Athens, “Sotiria” Hospital, 152 Mesogeion Ave, 11527 Athens, Greece; ^2^Hellenic Sports Research Institute, Olympic Sports Centre of Athens, 37 Kifisias Ave, Marousi, 15123 Athens, Greece

## Abstract

*Background*. Cognitive deterioration may impair COPD patient's ability to perform tasks like driving vehicles. We investigated: (a) whether subclinical neuropsychological deficits occur in stable COPD patients with mild hypoxemia (PaO_2_ > 55 mmHg), and (b) whether these deficits affect their driving performance. *Methods*. We recruited 35 stable COPD patients and 10 normal subjects matched for age, IQ, and level of education. All subjects underwent an attention/alertness battery of tests for assessing driving performance based on the Vienna Test System. Pulmonary function tests, arterial blood gases, and dyspnea severity were also recorded. *Results*. COPD patients performed significantly worse than normal subjects on tests suitable for evaluating driving ability. Therefore, many (22/35) COPD patients were classified as having inadequate driving ability (failure at least in one of the tests), whereas most (8/10) healthy individuals were classified as safe drivers (*P* = 0.029). PaO_2_ and FEV1 were correlated with almost all neuropsychological tests. *Conclusions*. COPD patients should be warned of the potential danger and risk they face when they drive any kind of vehicle, even when they do not exhibit overt symptoms related to driving inability. This is due to the fact that stable COPD patients may manifest impaired information processing operations.

## 1. Introduction

It is increasingly recognized that chronic obstructive pulmonary disease (COPD) is a multicomponent disease, but relatively little attention has been paid to its impact on neuropsychological function. Several studies have identified neuropsychological deficits in COPD patients [1–3]. The extent of this dysfunction appears to be related to the level of hypoxemia [4–8]. Subclinical cognitive deficits can even be detected in COPD patients with mild hypoxemia (PaO_2_ > 55 mm Hg) [9, 10].

Neuropsychological tests aim to provide standardized and objective measurements is the function of specific cognitive domains. The tasks, performed as part of the neuropsychological testing, often closely resemble mental challenges encountered in everyday life. One of the commonest mental challenges in everyday life is driving performance. The latter is a complex task highly dependent on the cognitive function, involving perceptual, motor, and decision making skills. Therefore, our hypothesis was that driving ability may be impaired even in stable COPD patients with mild hypoxemia.

 Road testing *per se* is the gold standard for assessing driving ability [11], but it is time consuming, expensive, and potentially hazardous. Simulators, which reproduce real driving [12, 13] conditions, are complex, very expensive, and not widely available. Nowadays, with advances in computer technology, various off-road neuropsychological tests have been developed to assess driving capacity. These tests are easier, obviously safer than on-road testing, and cheaper than using driving simulators. These tests measure an individual's ability to maintain attention, alertness, and proper reaction, the three key components of safe driving performance.

The aim of this work was to assess cognitive neuropsychological performance in a group of normal subjects and in a group of stable COPD patients with mild hypoxemia (i.e., PaO_2_ > 55 mm Hg) with a battery of pertinent neuropsychological tests, especially designed to evaluate driving-related ability.

Therefore, we conducted this preliminary study to investigate (a) whether the cognitive neuropsychological performance was impaired in COPD patients with subclinical levels of hypoxemia, that is, PaO_2_ > 55 mm Hg (primary outcome), and (b) whether this impaired performance was related to driving ability (secondary outcome). 

## 2. Methods

The population of the study consisted of 35 patients with COPD (26 males) and 10 normal subjects (8 males) who served as controls. The COPD patients referred to our laboratory for lung function testing. At study time, their clinical and functional state had been stable for at least four weeks. COPD severity was classified using postbronchodilator spirometric values according to the Global Initiative for Chronic Obstructive Lung Disease (GOLD) guidelines [14] (4 patients in stage I, 7 in stage II, 15 in stage III, and 9 in stage IV). Controls were never-smokers healthy volunteers with no medical history. The two groups were matched for age, gender, education, and intelligence quotient (IQ) as assessed by Raven's progressive matrices intelligence test (RPM) [15]. Subjects with history of neurological or psychiatric disease, head injury, uncorrected visual or acoustic impairment, shaking hands, chronic sedative intake, or alcohol abuse were excluded. Subjects with a history of asthma, allergic rhinitis, and BMI > 32 were also excluded. 

None of our patients participating in the study reported any symptoms and signs related to sleep apnoea syndrome. Although the Epworth Sleepiness Scale was not formally filled by the patients, all the pertinent questions were asked during the strict and detailed history taking. Therefore, a formal sleep study was not justified. On the other hand, any patients reporting suspicious symptoms or signs for OSAHS were excluded from the study.

 Randomly allocated, half of our patients have taken their daily dose of bronchodilator, but half of them have not taken it for at least 24 hours before neuropsychological testing. Any other medication was not allowed for at least 48 hours before testing. COPD patients were mildly hypoxemic (PaO_2_ > 55 mm Hg). Only 7 of them were hypercapnic (PaCO_2_ > 45 mm Hg). The characteristics of all subjects are presented in Table 1.

The study was approved by the local Medical Ethics Committee of Sotiria Hospital. All subjects gave their informed consent, and none of the participants received any financial compensation for their participation in the study.

### 2.1. Respiratory Function Tests

All control subjects and COPD patients underwent routine pulmonary function tests, that is, spirometry, static lung volumes, and lung diffusion capacity (DL_ CO _), according to the ATS/ERS guidelines [16–19]. The severity of chronic dyspnea was rated according to the modified Medical Research Council (mMRC) [20]. Arterial blood gases were measured only in COPD patients, and oxygen saturation (%SpO_2_) using a pulse oximeter was measured in all subjects. 

### 2.2. Neuropsychological Tests

**Figure 1:**
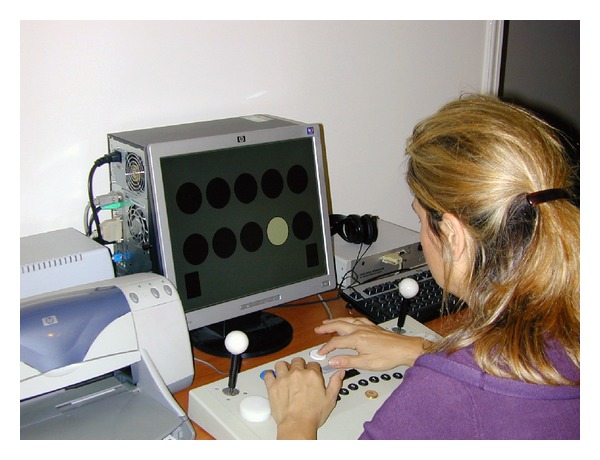
A subject performing neuropsychological testing for evaluating driving-related ability with the Vienna Test System.

Neuropsychological assessment took place on the same day after the pulmonary function tests. Every patient and healthy individual underwent an attention/alertness battery of tests for evaluating driving-related performance based on the Vienna Test System [21, 22] (http://www.schuhfried.co.at). Each test began with standardized instructions while the subject was comfortably seated in front of a computer's screen (Figure 1).

### 2.3. Reaction Time to Single Visual (RT-V) and Acoustic Stimuli (RT-A)

The subject places his forefinger on a detector and when a color flashlight (yellow light) appears on the screen he has to push a button 10 cm ahead in the fastest possible way (Figure 1). The use of a rest and a reaction key makes the splitting into reaction and motor time possible. So, two parameters are recorded: (a) the period of time between the flash light and the moment the subject takes his forefinger away from the detector-rest key (reaction time: RT-V), and (b) the period of time the subject takes his forefinger away from the detector and pushes the button (motor time: MR-V). The sum of the two times above is the total reaction time (total RT-V). Totally, 28 stimuli are presented, and the test duration is 7 minutes.

Reaction time to acoustic stimuli is performed in the same way, except that the flashlight is replaced by a sound presented to the subject via headphones. The total reaction time (total RT-A) is the sum of the reaction time (RT-A) and motor time (MR-A) to acoustic stimuli. 

### 2.4. Selective Attention Test (SA)

It is a test for the assessment of concentration. The program presents four geometrical shapes on the top of the screen and asks from the subject to compare these shapes with a geometrical shape shown at the bottom of the screen. The number of given tasks is 80, and the duration of the test is 20 min.

### 2.5. Permanent Attention Test (PA)

This test assesses reaction under stress. The subject has to match quickly color figures with the equivalent color buttons on a keyboard, react to acoustical signs of high or low frequency by pushing predetermined corresponding buttons, and press foot pedals when the figure of a foot pedal appears on the screen. The total number of presented stimuli is 150, and the test duration is 30 min. 

### 2.6. Tachistoscopic Traffic Test (TAVTMB)

This test assesses visual perception. The subject is confronted with 20 pictures of traffic situation for 1 second each. Then, he has to indicate what he has seen in the picture. The test duration is 10 min.

According to the results of the tests, the subject is classified in a percentile of preexisting normative values of age-matched controls. Normative data exists from general adult population from all over Europe, of different social-economical and educational groups, with age distribution of 18–80 years old (http://www.schuhfried.at).

Control subjects and COPD patients were also evaluated according to Raven's intelligence test [15]. This IQ test consists of 60 items. Each item contains a figure with a missing piece and alternative pieces to complete the figure, only one of which is correct. The raw score is typically converted to a percentile rank by using the appropriate norms. Subjects had at their disposal 30 minutes to complete the test. Subjects with IQ scores <50% ile were not acceptable in order to avoid wrong answers in all other neuropsychological tests because of the difficulty to understand them.

In addition, both patients and control subjects underwent ophthalmologic and audiologic examinations before the tests to exclude hearing or visual deficits compromising the reliability and validity of neuropsychological testing.

According to the European diagnostic criteria for the assessment of driving ability based on the Vienna Test System, subjects have to pass all the tests in order to be classified as having adequate driving-related ability and obtain a professional driving licence. An expert in traffic psychology evaluates patients' performance on all the tests and identifies who are fit or unfit to drive [16].

### 2.7. Statistical Analysis

The statistical analysis and related graphs were performed using SigmaStat V3.5 and SigmaPlot V10.0 (Jandel Scientific, CA, USA). For comparisons between groups, the Student's unpaired *t*-test was used. If there was no normality in the distribution in any of our parameters, a Mann-Whitney *U*-test for unpaired values was used. Where appropriate, Spearman correlation analysis, linear regression analysis, one-way ANOVA on ranks, multiple regression, and forward stepwise regression were used. 

## 3. Results

All subjects' anthropometric and lung function data are shown in Table 1. Controls and COPD patients had comparable age, height, weight, BMI, and IQ. COPD patients were significantly different to controls in pulmonary function data and oxygen saturation.

Table 2 summarises the results obtained in the attention/alertness battery of tests for the two groups. All the results are expressed as percentile of preexisting normative values. The faster the performance, the higher the score. Patients had significantly longer reaction and motor times in response to visual and acoustic stimuli and so, lower scores. They also presented significantly reduced visual perception (TAVTMB). Patients tended to score worse than controls for selective and permanent attention test, but this difference did not reach statistical significance. So, COPD patients scored worse than healthy volunteers in five of the seven neuropsychological tests for assessing driving-related performance. Among 35 COPD patients, only 13 successfully completed all the tests, and these 13 were classified as safe drivers. Among 10 controls, only 2 failed to complete all the tests and were classified as unsafe drivers (*P* = 0.029). 

**Figure 2:**
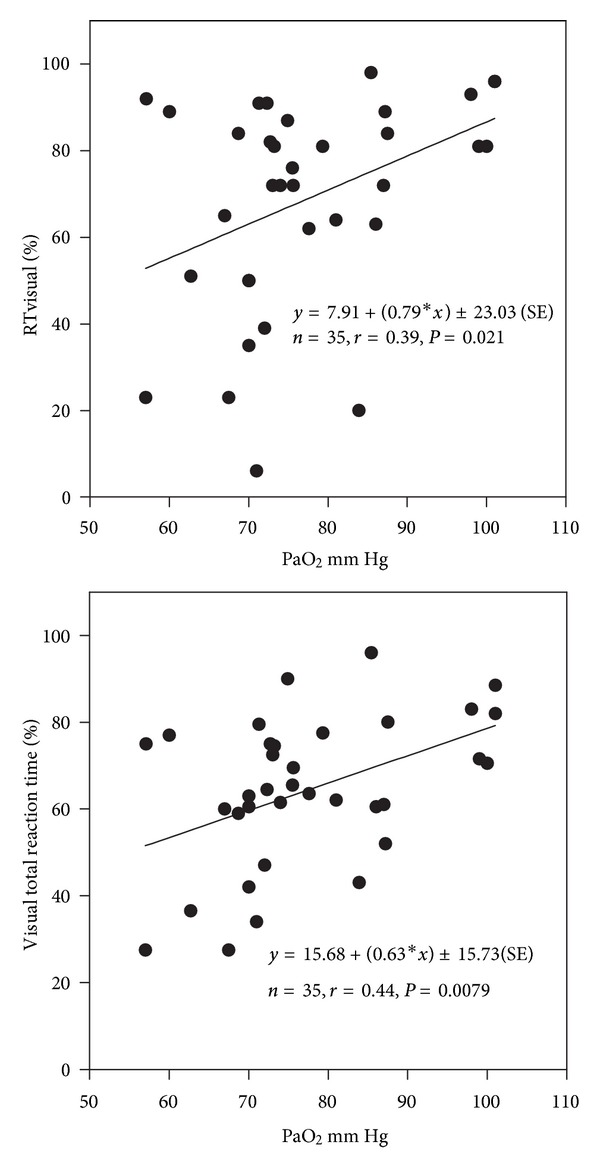
Relationship of PaO_2_ to simple reaction time (RT-V%) and total reaction time (total RT-V%) to visual stimuli.

**Figure 3:**
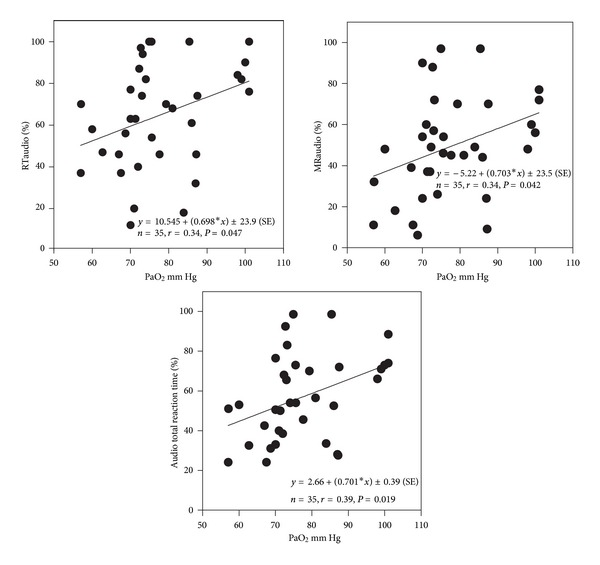
Relationship of PaO_2_ to simple reaction time (RT-A%), motor reaction time (MR-A%), and total reaction time (total RT-A%) to audio stimuli.

**Figure 4:**
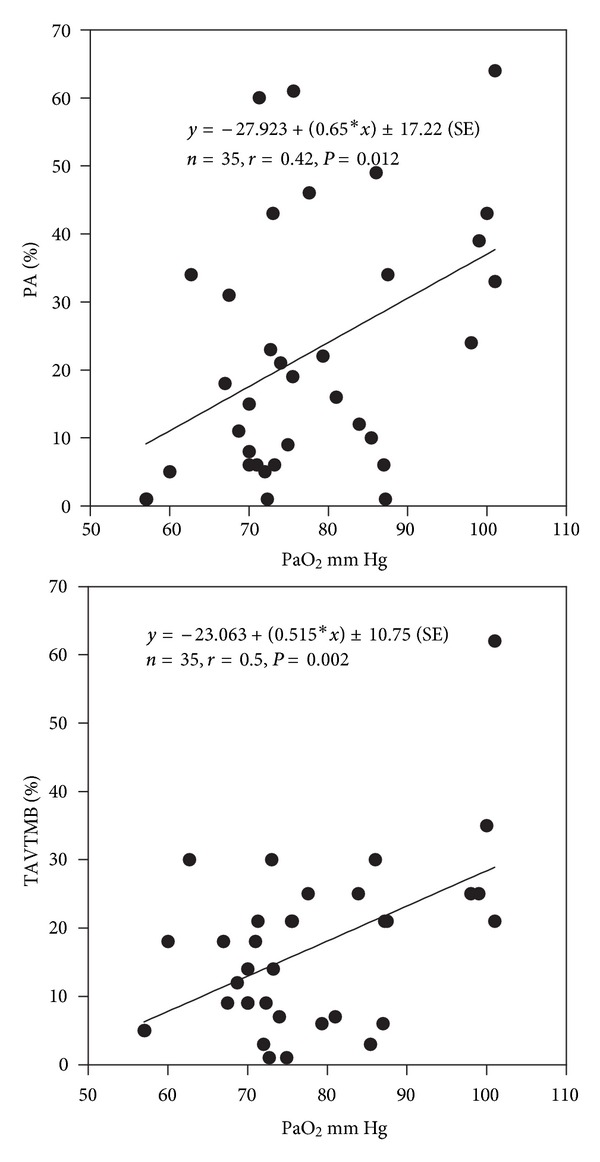
Relationship of PaO_2_ to permanent attention (PA%) and tachistoscopic traffic (TAVTMB%) tests.

**Figure 5:**
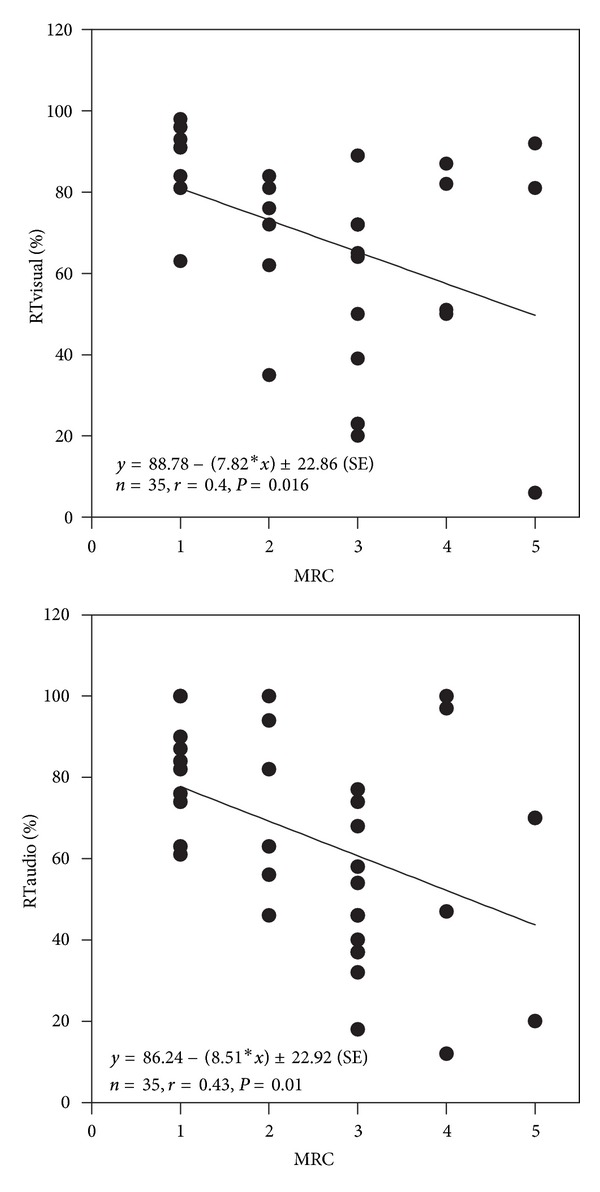
Relationship of dyspnea severity according to the modified Medical Research Council (mMRC) to reaction time to visual (RT-V%) and audio (RT-A%) stimuli.

**Figure 6:**
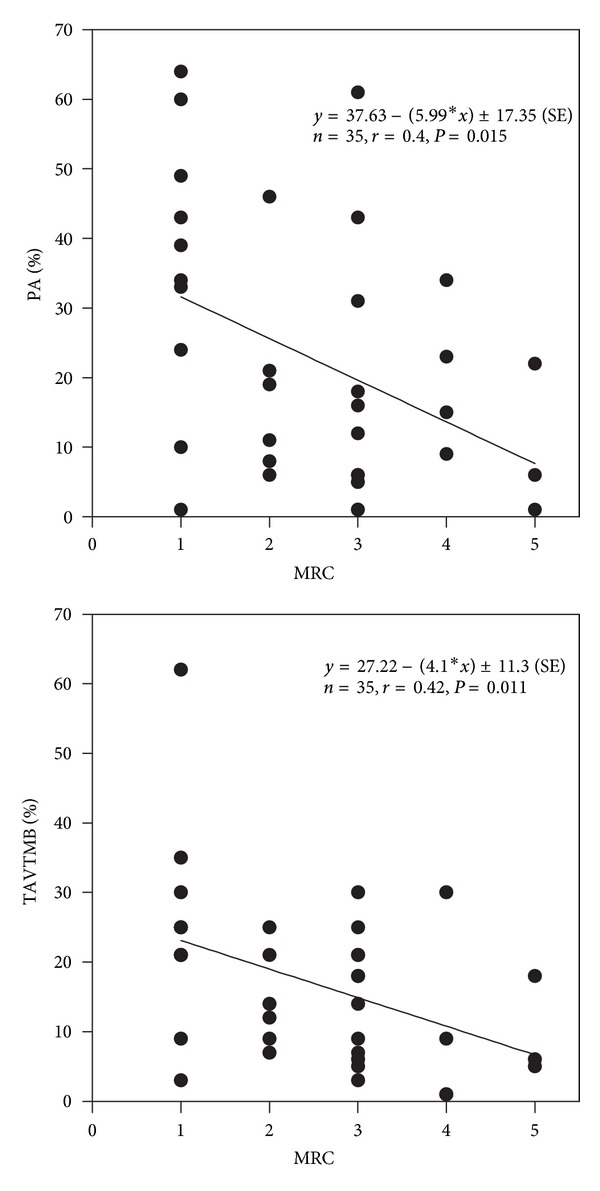
Relationship of dyspnea severity according to the modified Medical Research Council (mMRC) to permanent attention (PA%) and tachistoscopic traffic (TAVTMB%) tests.

**Figure 7:**
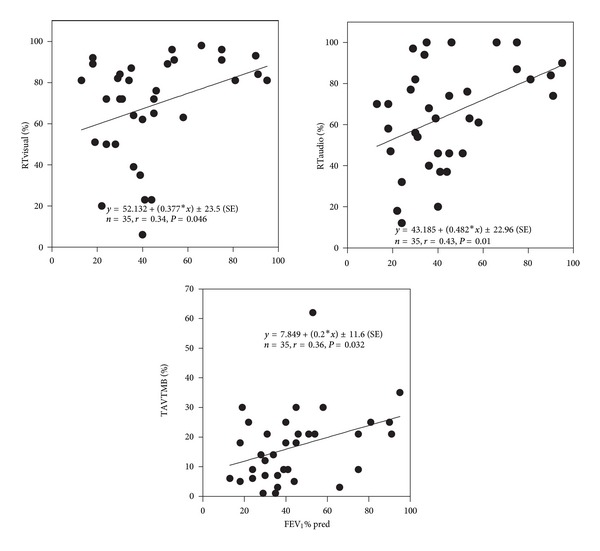
Relationship of FEV_1_% pred to reaction time to visual (RT-V%) stimuli, reaction time to audio (RT-A) stimuli, and tachistoscopic traffic test (TAVTMB%).

All neuropsychological tests were significantly (*P* < 0.05) correlated with PaO_2_, except for the selective attention test (SA) (Figures 2, 3, and 4). SA test was correlated only with a lower IQ. The severity of dyspnea rated according to the mMRC score seems to influence significantly the performance on RT-V, RT-A, PA, and TAVTMB tests (Figures 5 and 6). The performance on the same tests, except for the PA test, was significantly correlated with FEV_1_% pred (Figure 7). The effect of FEV_1_ especially on visual perception (TAVTMB) can be verified if we divide patients into GOLD stages. In stage I, the median value of visual perception is 25%, the same as for the control group. In stage II, it is 21%, in stage III, 12%, and in stage IV, 9%. There is a statistically significant difference among the groups (*P* < 0.003).

With simple correlations, we could not find any effect of PaCO_2_ on psychomotor tests. To further investigate the real effect of PCO_2_ on psychomotor performance, we divided our patients into two groups: those in whom PCO_2_ was normal (≤45 mm Hg) (*n* = 28) and those whose PCO_2_ was >45 mm Hg and were hypercapnic (*n* = 7). The two groups had similar scores on the tests except for the motor reaction time to visual and acoustic stimuli. Hypercapnic COPD patients scored lower than nonhypercapnic on these tests, and this difference remained significant after adjustment for potential confounders such as PaO_2_ and age.

As we have already mentioned, randomly allocated, half of our patients (*n* = 17) have taken their daily dose of an inhaled drug (6 patients have taken a b_2_ bronchodilator and 11 a combination of a b_2_ bronchodilator and anticholinergic or corticosteriod) at least one hour before testing, and half of them have not taken it, at least 24 hours before testing (*n* = 18). The two groups showed no significant differences on the neuropsychological tests. So, it appears that bronchodilators do not influence driving-related psychomotor performance.

Finally, we divided the 35 COPD patients into two groups: those who have been accepted (*n* = 13 patients) and those who have been rejected (*n* = 22 patients) as safe drivers. The two groups showed statistically significant differences for PaO_2_, SpO_2_, FEV_1_, FVC, IC, RV, and DLCO. They also showed statistically different scores in SA, total RT-V, PA, and TAVTMB tests (Table 3). They were not matched for age and IQ, and after correction for age and IQ with logistic regression, they remained different in the last two tests, that is, permanent attention and visual perception. 

## 4. Discussion

In this study, we have shown that, except of the well-known cognitive dysfunction in severe hypoxemic patients [23–25], cognitive performance is also impaired in mildly hypoxemic COPD patients when compared to normal subjects matched for age, education level, and IQ. One of the main practical effects of this deterioration is the impairment of a patient's ability to perform tasks requiring increased vigilance and alertness like driving any kind of vehicle [26]. To the best of our knowledge, there are no reports dealing with the problem of impaired driving ability in COPD patients by using especially designed computer-based neuropsychological tests [27].

There are sparse publications and controversial reports for COPD patients with mild hypoxemia [9, 10]. The practical effect of the cognitive deterioration to the daily lives of these patients is still not known. Driving is an essential part of everyday life for most people, and the withholding of a private or professional driving licence has major implications for social functioning and employment. According to traffic psychology, accident proneness has strong relationships with a number of perceptual, cognitive, and motor skills. In our study, COPD patients demonstrated markedly delayed reaction times to visual or acoustic stimuli and impairments in motor activity and perceptive speed in traffic situations. These Subclinical neuropsychological deficits may explain the worse driving-related performance of COPD patients compared to normal subjects. More than half of our COPD patients (22/35) were classified as unsafe drivers based on failing to at least one from a battery of neuropsychological tests pertinent to any driving situation.

The explanation for the impaired functioning in COPD patients can be the mildly low levels of blood oxygenation, given the fact that the brain is the most sensitive organ to oxygen lack. This level of hypoxemia could lead to mild-to-moderate inefficiencies in neural functioning and thus to the modest subclinical impairment. In this study, we have included mildly hypoxemic patients (PaO_2_ > 55 mm Hg); therefore, it is possible that a not fully normal PaO_2_ or at the lower limits of normal (between 55–80 mm Hg) still leads to an impaired subclinical cognitive performance.

In addition, these patients usually have nocturnal desaturation or hypoxemia during sleep [27, 28], chronic pulmonary disease could enhance vascular disease, leading to reduced cerebral blood flow and oxygen consumption, even in normoxemic COPD patients, and COPD *per se* could lead to an acceleration of the aging process so that brain functions are impaired in a fashion similar to that seen in the elderly. This process could lead to a reduction in cortical neuronal density and a subsequent less efficient performance on neuropsychological tests. All these factors could be at work and have additive effects [29].

Impairments of cognitive performance in patients with COPD can be predicted on the basis of the severity of the disease. The partial pressure of arterial oxygen and the degree of pulmonary impairment may be major factors contributing to cognitive deficit among COPD patients. The partial pressure of arterial dioxide seems to affect the performance on motor reaction time to visual or acoustic stimuli, which require motor muscle activity. The muscle weakness that is frequently seen in patients with COPD may explain the previous observation [30]. However, the permanent attention test, another test requiring motor muscle performance, does not seem to be influenced by PaCO_2_. So, impairments in motor ability cannot simply be explained by weak muscle activity. Irrespective of the cause, these deficits may have negative impact on driving performance of any kind of vehicle ranging from a bicycle to heavy lorries, in real traffic settings.

Possible limitations of the present study lie in whether these findings represent state rather than trait effects, which appears to be reasonable target for future research. In this sense, future research should replicate the main findings in independent samples as well as further explore whether the findings are associated in task-specific manner or across tasks.

Driving fitness may be assessed with reasonable accuracy using off-road tests minimizing the expense and risk associated with on road assessment. However, computer-based testing does not provide the real changes that occur when turning a steering wheel and the vehicle changes course. Also, missing in the laboratory environment is the subject's knowledge that the consequences of driving control responses affect his/her own safety.

Although there are disadvantages in computer-based testing, these are easy and simple tests that might be useful for giving insight about driving performance in COPD patients and make an important contribution to transport safety. These preliminary data need to be confirmed with further studies before simple computer-based testing can be used to decide whether or not an individual is safe to drive in every day life. 

We conclude that probably stable COPD patients should be warned of the possible danger and risk they face when they drive any kind of vehicle, even when they do not exhibit overt symptoms related to driving ability.

## Figures and Tables

**Table 1:** Anthropometric characteristics and respiratory function data of normal subjects and COPD patients.

Parameters	Normal subjects (*n* = 10)	COPD patients (*n* = 35)	*P* value
Age, (yrs)	55 (5)	59 (7)	NS
Gender, M/F	8/2	26/9	NS
Ht, (m)	1.7 (0.05)	1.7 (0.07)	NS
Wt, (kg)	78 (8)	77 (14)	NS
Wt, (% pred)	109 (10)	106 (15)	NS
BMI	27.4 (2.7)	26.5 (3.7)	NS
FVC, (% pred)	105 (12)	86 (20)	*P* = 0.007
FEV_1_, (% pred)	100 (11)	45 (22)	*P* < 0.001
FEV_1_/FVC, %	77 (4)	40 (14)	*P* < 0.001
IC, (% pred)	103 (18)	81 (17)	*P* < 0.001
TLC, (% pred)	93 (8)	100 (15)	NS
FRC, (% pred)	89 (12)	120 (0.0)	*P* = 0.002
RV, (% pred)	74 (14)	123 (42)	*P* < 0.001
DL_CO_, (% pred)	103 (12)	60 (22)	*P* < 0.001
PaO_2_ (mm Hg)		77 (12)	
PaCO_2_ (mm Hg)		41 (6)	
SpO_2_ %	98 (97–99)	95 (94–96)	*P* < 0.001
IQ (% ile)	90 (75–95)	80 (63–95)	NS

Values are mean (SD) or median (range).

Abbreviations: Ht: height; Wt: weight; BMI: body mass index; SpO_2 _%: arterial oxygen saturation measured with pulse arterial oximeter; IQ: intelligent quotient; *P* ≤ 0.05, statistically significant; NS: nonsignificant.

**Table 2:** Driving-related neuropsychological testing performance data in normal subjects and COPD patients.

Parameters	Normal subjects (*n* = 10)	COPD patients (*n* = 35)	*P* value
SA, (% ile)	32.5 (19–39)	23.9 (9.3–34.8)	NS
RT-V, (% ile)	89 (82–89)	76 (53.8–88.5)	*P* = 0.035
MR-V, (% ile)	79.2 (12.2)	59.8 (18.7)	*P* = 0.004
Total RT-V, (% ile)	82 (79–87.5)	64.5 (59.3–76.5)	*P* < 0.0001
RT-A, (% ile)	84.5 (70–97)	68 (46–83.5)	*P* = 0.035
MR-A, (% ile)	72.6 (22)	49.2 (24.7)	*P* = 0.01
Total RT-A, (% ile)	78.3 (14.2)	56.9 (21.6)	*P* = 0.0052
PA, (% ile)	26.5 (17–38)	18 (6–34)	NS
TAVTMB, (% ile)	25 (21–62)	18 (7–24)	*P* = 0.003
Driving ability, accepted/rejected	8/2	13/22	*P* = 0.029

Values are mean ± SD or median (range).

Abbreviations: SA: selective attention; RT-V: reaction time to visual stimuli; MR-V: motor time to visual stimuli; Total RT-V: the sum of reaction and motor time to visual stimuli; RT-A: reaction time to audio stimuli; MR-A: motor time to audio stimuli; Total RT-A: the sum of reaction and motor time to audio stimuli; PA: permanent attention; TAVTMB: tachistoscopic traffic test; *P* ≤ 0.05, statistically significant; NS: nonsignificant.

**Table 3:** Characteristics and attention/alertness performance data for COPD patients who have been accepted and those who have been rejected as safe drivers.

	Rejected (*n* = 22)	Accepted (*n* = 13)	*P*
AGE, (yrs)	61.5 (6.6)	55.7 (6.6)	0.017
Ht, (m)	1.68 (0.1)	1.73 (0.1)	0.042
Wt, (% pred)	104.8 (92–117)	111 (96–122)	NS
BMI	26.1 (3.5)	27.1 (4)	NS
FVC, (% pred)	79.7 (18.7)	96.8 (18.2)	0.013
FEV_1_, (% pred)	35.4 (14.9)	59.8 (24.5)	<0.001
FEV_1_/FVC, %	45.6 (13)	63.2 (20.5)	0.004
IC, (% pred)	75.6 (13.4)	90.2 (20)	0.015
TLC, (% pred)	101 (16.6)	98.4 (10.4)	NS
RV, (% pred)	133.4 (47.6)	104.1 (20.3)	0.044
RV/TLC (%)	47.9 (10.6)	36.5 (8.9)	0.003
DLCO, (% pred)	50.6 (15.3)	74.8 (24)	<0.001
PaO_2_ (mm Hg)	72.2 (68.7–79.3)	86 (74.9–99.3)	0.005
PaCO_2_ (mm Hg)	41.6 (6)	38.7 (4.13)	NS
SaO_2_, (%)	94.2 (2.3)	96.4 (1.9)	0.006
SpO_2_, (%)	93.5 (2.4)	96.5 (2.0)	<0.001
mMRC, grade	3 (2–4)	1 (1–2.5)	0.002
IQ (% ile)	75 (50–85)	90 (80–95)	0.009
SA, (% ile)	19.5 (17.1)	36 (23.5)	0.021
RT-V, (% ile)	63.3 (27.9)	78.3 (14.1)	NS
MR-V, (% ile)	57.5 (20.8)	63.7 (14.5)	NS
Total RT-V, (% ile)	61.3 (47–75)	71.5 (65–80.5)	0.047
RT-A, (% ile)	59.5 (27.4)	73.2 (18.2)	NS
MR-A, (% ile)	47.2 (28.9)	52.6 (15.8)	NS
Total RT-A, (% ile)	53.4 (24.3)	62.9 (15)	NS
PA, (% ile)	8.5 (5–16)	43 (33.8–51.8)	<0.001
TAVTMB, (% ile)	9 (5–14)	25 (21–30)	<0.001

Values are mean (SD) or median (range).

Abbreviations as in Table 3.

## Abbreviations

ATS: American Thoracic Society
BMI: Body mass index
COPD: Chronic obstructive pulmonary disease
DLCO: Lung diffusion capacity
FEV_1_: Forced expiratory volume in one second
FRC: Functional residual capacity
GOLD: The global initiative for chronic obstructive lung disease
IC: Inspiratory capacity
IQ: Intelligence quotient
mMRC: modified Medical Research Council
MR-A/MR-V: Motor reaction time to audio/visual stimuli
PA: Permanent attention
RT-A/RT-V: Reaction time to audio/visual stimuli
RPM: Raven's progressive matrices
SA: Selective attention
TAVTMB: Tachistoscopic traffic test
TLC: Total lung capacity.

## References

[1] Incalzi RA, Gemma A, Marra C, Muzzolon R, Capparella O, Carbonin P (1993). Chronic obstructive pulmonary disease: an original model of cognitive decline. *American Review of Respiratory Disease*.

[2] Klein M, Gauggel S, Sachs G, Pohl W (2010). Impact of chronic obstructive pulmonary disease (COPD) on attention functions. *Respiratory Medicine*.

[3] Hung WW, Wisnivesky JP, Siu AL, Ross JS (2009). Cognitive decline among patients with chronic obstructive pulmonary disease. *American Journal of Respiratory and Critical Care Medicine*.

[4] Fix AJ, Golden CJ, Daughton D (1982). Neuropsychological deficits among patients with chronic obstructivepulmonary disease. *International Journal of Neuroscience*.

[5] Grant I, Heaton RK, McSweeney AJ (1982). Neuropsychologic findings in hypoxemic chronic obstructive pulmonary disease. *Archives of Internal Medicine*.

[6] Grant I, Prigatano GP, Heaton RK, McSweeny AJ, Wright EC, Adams KM (1987). Progressive neuropsychologic impairment and hypoxemia. Relationship in chronic obstructive pulmonary disease. *Archives of General Psychiatry*.

[7] Krop HD, Block AJ, Cohen E (1973). Neuropsychologic effects of continuous oxygen therapy in chronic obstructive pulmonary disease. *Chest*.

[8] Heaton RK, Grant I, McSweeny AJ (1983). Psychologic effects of continuous and nocturnal oxygen therapy in hypoxemic chronic obstructive pulmonary disease. *Archives of Internal Medicine*.

[9] Prigatano GP, et A (1983). Neuropsychological test performance in mildly hypoxemic patients with chronic obstructive pulmonary disease. *Journal of Consulting and Clinical Psychology*.

[10] Liesker JJW, Postma DS, Beukema RJ (2004). Cognitive performance in patients with COPD. *Respiratory Medicine*.

[11] Mazza S, Pepin J-L, Naegele B (2006). Driving ability in sleep apnoea patients before and after CPAP treatment: evaluation on a road safety platform. *European Respiratory Journal*.

[12] Juniper M, Hack MA, George CF, Davies RJO, Stradling JR (2000). Steering simulation performance in patients with obstructive sleep apnoea and matched control subjects. *European Respiratory Journal*.

[13] Orth M, Duchna H-W, Leidag M (2005). Driving simulator and neuropsychological testing in OSAS before and under CPAP therapy. *European Respiratory Journal*.

[14] Gold Report http;//www.goldcopd.org/.

[15] Raven JC, Court JH, Raven J (1976). *Manual for Raven’s Progressive Matrices*.

[16] (2007). Directive 2007/59/EC of the European Parliament and the council on the certification of train drivers operating locomotives and trains on the railway system in the Community. *Official Journal of the European Union*.

[17] Miller MR, Hankinson J, Brusasco V (2005). Series “ATS/ERS TASK FORCE: standardisation of lung function testing” standardisation of spirometry. *European Respiratory Journal*.

[18] Wanger J, Clausen JL, Coates A (2005). Series “ATS/ERS TASK FORCE: standardisation of lung function testing” Standardisation of the measurement of lung volumes. *European Respiratory Journal*.

[19] Maclntyre N, Crapo RO, Viegi G (2005). Series “ATS/ERS TASK FORCE: standardisation of lung function testing” Standardisation of the single-breath determination of carbon monoxide uptake in the lung. *European Respiratory Journal*.

[20] Quanjer PhH (1993). Standardized lung function testing. Report Working Party “Standardization of Lung Function Tests”, European Community for Coal and Steel. *European Respiratory Journal*.

[21] Bestall JC, Paul EA, Garrod R, Garnham R, Jones PW, Wedzicha JA (1999). Usefulness of the Medical Research Council (MRC) dyspnoea scale as a measure of disability in patients with chronic obstructive pulmonary disease. *Thorax*.

[22] Schuhfried G (2001). *Computer-Aided Procedures for Ability and Personality Diagnostics. Catalogue*.

[23] Alchanatis M, Zias N, Deligiorgis N, Amfilochiou A, Dionellis G, Orphanidou D (2005). Sleep apnea-related cognitive deficits and intelligence: an implication of cognitive reserve theory. *Journal of Sleep Research*.

[24] Hynninen KMJ, Breitve MH, Wiborg AB, Pallesen S, Nordhus IH (2005). Psychological characteristics of patients with chronic obstructive pulmonary disease: a review. *Journal of Psychosomatic Research*.

[25] Incalzi AR, Chiappini F, Fuso L, Torrice MP, Gemma A, Pistelli R (1998). Predicting cognitive decline in patients with hypoxaemic COPD. *Respiratory Medicine*.

[26] Incalzi RA (1997). Verbal memory impairment in COPD: its mechanisms and clinical relevance. *Chest*.

[27] Orth M, Diekmann C, Suchan B (2008). Driving performance in patients with chronic obstructive pulmonary disease. *Journal of Physiology and Pharmacology*.

[28] Calverley PMA, Brezinova V, Douglas NJ (1982). The effect of oxygenation on sleep quality in chronic bronchitis and emphysema. *American Review of Respiratory Disease*.

[29] Cormick W, Olson LG, Hensley MJ, Saunders NA (1986). Nocturnal hypoxaemia and quality of sleep in patients with chronic obstructive lung disease. *Thorax*.

[30] Gibson GE, Pulsinell W, Blass JP, Duffy TE (1981). Brain dysfunction in mild to moderate hypoxia. *American Journal of Medicine*.

